# Knowledge, attitudes, practices, and self-efficacy of the Chinese public regarding cardiopulmonary resuscitation: an online cross-sectional survey

**DOI:** 10.3389/fpubh.2024.1341851

**Published:** 2024-02-29

**Authors:** Huiming Gao, Xiaohui Liu, Zhixia Jiang, Shiming Huang, Xiaoying Pan, Jianmei Long, Qingqing Tong, Li Li, Manhong Zhou, Rujun Hu

**Affiliations:** ^1^Department of Nursing, Affiliated Hospital of Zunyi Medical University, Zunyi, Guizhou, China; ^2^School of Nursing, Zunyi Medical University, Zunyi, Guizhou, China; ^3^Guizhou Nursing Vocational College, Guiyang, Guizhou, China; ^4^Department of Emergency, Affiliated Hospital of Zunyi Medical University, Zunyi, Guizhou, China; ^5^Department of Emergency, Kweichow Moutai Hospital, Renhuai, Guizhou, China; ^6^Department of Critical Care Medicine, Affiliated Hospital of Zunyi Medical University, Zunyi, Guizhou, China

**Keywords:** cardiopulmonary resuscitation, knowledge, attitude, practice, self-efficacy

## Abstract

**Objective:**

To evaluate the current status of Chinese public’s knowledge, attitudes, practices (KAP) and self-efficacy regarding cardiopulmonary resuscitation (CPR), and to analyze the factors that influence KAP and self-efficacy.

**Methods:**

An online cross-sectional survey was conducted from February to June 2022 in Mainland China via a self-designed self-filled questionnaire. Potential participants were recruited through WeChat by convenience sampling and snowball sampling methods. Descriptive and quantitative analyses were used for statistical analysis.

**Results:**

The survey included 4,450 participants from 31 provinces, autonomous regions, or municipalities across Mainland China, aged 18 or above. The public’s average understanding (clear and very clear) of the knowledge regarding CPR was 67.4% (3,000/4,450), with an average proportion of positive attitudes at 96.8% (4,308/4,450). In practice, the average proportion of good practices was 92.8% (4,130/4,450), while the percentage of good self-efficacy averaged at 58.9% (2,621/4,450), only 42.4% (1,885/4,450) of the participants had confidence in the correct use of automated external defibrillator (AED). Pearson correlation analysis showed a significantly positive correlation among knowledge, attitude, practice, and self-efficacy (*p* < 0.01). Multiple linear regression analysis revealed that several factors have a significant influence on the public’s CPR KAP and self-efficacy, including ever having received CPR training (*p* < 0.001), hearing about AED (*p* < 0.001), performing CPR on others (*p* < 0.001), hearing about CPR (*p* < 0.001), occupation (*p* < 0.001), personal health status (*p* < 0.001), education level (*p* < 0.001), gender (*p* < 0.001), and encountering someone in need of CPR (*p* = 0.021).

**Conclusion:**

The Chinese public demonstrates good knowledge of CPR, positive attitude, and high willingness to perform CPR. However, there is still room for improvement in the mastery of some professional knowledge points related to CPR and AED. It should be noted that knowledge, attitude, practice, and self-efficacy are interrelated and influence each other. Factors such as prior CPR training, hearing about AED, having performed CPR before, hearing about CPR, occupation, personal health status, education level, gender, and having encountered someone in need of CPR have a significant impact on the public’s KAP and self-efficacy.

## 1 Introduction

Out-of-hospital cardiac arrest (OHCA) is a significant cause of out-of-hospital deaths and has emerged as a major public health issue across the globe. The incidence of OHCA ranges from 40.8 to 100.2 per 100,000 person-years worldwide (1). The latest report indicates that the overall incidence of OHCA in China is 97.1 per 100,000 person-years, showing an upward trend compared to 10 years ago (2). More than 230 million people in China have cardiovascular disease, and each year, 550,000 individuals experience cardiac arrest, with a survival rate estimated to be 1.2% (2, 3).

Initiating bystander cardiopulmonary resuscitation (CPR) early is a critical contributing factor in improving survival rates for OHCA (4, 5). Studies have demonstrated that patients who received CPR from bystanders had a 2.6 times higher 30-day survival rate than those who did not receive CPR (5). However, the average rate of bystander CPR performed in China is only 17% (2), whereas in the USA, England, France, and Europe, it is approximately 40.2, 55.2, 51, and 58%, respectively (6–9). The low CPR rate means that patients experiencing sudden OHCA do not receive timely rescue, which is concerning.

The experience of other national OHCA survival programs has demonstrated that increasing bystander CPR can improve overall survival (10), and training more individuals is an effective approach to boost bystander CPR (11). However, China’s general training rate of 5.74 to 25.6% (12–14) is significantly lower than that of the US (41.9%), France (40%) and Scotland (52%) (3, 15, 16). To address this gap and bridge the divide between China and other developed countries in terms of public CPR training rates, in 2016, the Chinese Medical Association and the Chinese Association of Research Hospitals jointly launched the “Health Project Plan for Popularizing CPR into 100 Million Households”: 525 + I Love My Family Project. The initiative encourages each medical staff to teach CPR techniques and related knowledge to 5 families using “point to area” and “snowball” techniques, with the goal of popularizing CPR among 200 million people within 5 years (17). Furthermore, in 2019, the Chinese government released the “Healthy China Action Plan (2019–2030),” which encourages mass training in first aid and aims to increase the proportion of personnel with first aid training certificates to at least 3% by 2030 (18).

With the increasing awareness of first aid among the public, more and more people have realized the importance of CPR. Although there have been some research studies on knowledge, attitudes, practices (KAP) and self-efficacy of CPR among the Chinese population in recent years, most of these studies were conducted in specific localities and focused on particular groups such as students, community residents, healthcare professionals, and so on. Furthermore, there were significant differences in the results obtained from different studies (14, 19–22). To our knowledge, there is currently no research report on the overall state of KAP and self-efficacy regarding CPR among the Chinese public. In order to gain a better understanding of the latest status of the Chinese public’s KAP and self-efficacy regarding CPR, we conducted a nationwide survey and analyzed the factors influencing KAP and self-efficacy, as well as the correlations among them. This information will provide a useful reference for further enhancing the CPR skills of the general public.

## 2 Methods

### 2.1 Study design and participants

This study utilized a cross-sectional online survey conducted on a specialized questionnaire survey platform (Wenjuanxing, https://www.wjx.cn/) in China between February and June 2022. Convenience sampling and snowball sampling methods were used to select potential respondents. The researchers invited WeChat friends to complete an electronic questionnaire through a web link or a QR code posted on WeChat. Respondents voluntarily completed the questionnaire and forwarded it to their WeChat friends. All participants were required to obtain informed consent before completing the questionnaire. If respondents declined to fill out the questionnaire, they automatically exited the survey. Inclusion criteria were being 18 years old or older, able to use a smartphone, and able to comprehend the content of the questionnaire. Exclusion criteria were being unable to fill in the questionnaire due to illness or other reasons.

The sample size for this study was calculated using the formula: N = (μ_α/2_/δ)^2^ P (1−P). N represents the sample size; μ_α/2_ refers to the statistic of 1.96 for a two-sided test with a confidence interval of 95%, δ represents the permissible error, and P represents CPR training rate of Chinese public. Based on a general training rate of 5.74 to 25.6% (12–14), an permissible error (δ) of 0.03, α = 0.05, 1 − β = 0.9, and an expected 20% nonresponse rate, we estimated the sample size to be between 278 and 976 participants.

This study adheres to the requirements of medical ethics and has been reviewed and approved by the Ethics Committee of the Affiliated Hospital of Zunyi Medical University (Approval No. KLL-2022-177).

### 2.2 Instrument

To achieve the research objectives, the research team initially developed a draft questionnaire based on the “2020 American Heart Association Guidelines for Cardiopulmonary Resuscitation and Emergency Cardiovascular Care” (4), our team’s previous research (23) and other related literature. Thereafter, seven experts in emergency medicine and emergency nursing from the Affiliated Hospital of Zunyi Medical University were invited to conduct two rounds of expert consultation to refine and improve the questionnaire. After two rounds of expert consultation to form the first draft of the questionnaire, 30 individuals from various sociodemographic backgrounds were selected through convenience sampling to complete the pre-survey, and their feedback was used to evaluate the questionnaire’s comprehensibility, applicability, and time required to complete it. Based on the feedback from participants in the pre-survey, further modifications were made to produce the final version of the questionnaire.

The final questionnaire consisted of 52 questions in five sections, including general information, CPR knowledge, CPR attitudes, willingness to practice CPR, and self-efficacy, as shown in the Supplementary material S1 of this manuscript. The Likert five-point rating scale was used for all sections except for general information. The knowledge section of the questionnaire consisted of 12 items, each with response options ranging from “very clear” to “very unclear” and scored from 5 to 1 points, resulting in a total score of 60 points. A higher score indicated a greater level of knowledge mastery. The attitude section comprised 10 items, with response options ranging from “strongly agree” to “strongly disagree” and scored from 5 to 1 points, resulting in a total score of 50 points. A higher score indicated a more positive attitude. The behavior section contained 4 items, with response options ranging from “strongly willing” to “strongly unwilling” and scored from 5 to 1 points, resulting in a total score of 20 points. A higher score indicated a stronger behavioral intention. Finally, the self-efficacy section consisted of 4 items, with response options ranging from “extremely confident” to “not confident at all” and scored from 5 to 1 points, resulting in a total score of 20 points. A higher score indicated better self-efficacy.

The content validity index (CVI) of the questionnaire was confirmed by 7 experts from the fields of emergency medicine and emergency nursing. Prior to the formal survey, we conducted a pilot study with 50 participants, excluding the 30 participants mentioned earlier who participated in the evaluation questionnaire’s comprehensibility, to evaluate the internal consistency reliability (Cronbach’s α). The final CVI was 0.95, and the Cronbach’s α value was 0.92, which suggests that this questionnaire is reliable and valid.

### 2.3 Data collection

The self-designed questionnaire was uploaded onto an online survey platform (Wenjuanxing, https://www.wjx.cn/), and the link or QR code to the questionnaire webpage was distributed to potential respondents via WeChat, using convenience and snowball sampling methods. Prior to completing the questionnaire, respondents were presented with online informed consent, which enabled them to decide whether to participate in the survey. If a respondent chose not to complete the questionnaire, they were automatically exited from the survey. We encouraged participants to assist in sharing the questionnaire link with their WeChat friends and social circles to expand the reach of the survey. All questions were mandatory, and respondents had to complete all questions before submission. Furthermore, each electronic device was restricted to one response to prevent duplicate submissions.

### 2.4 Statistical analysis

We analyzed the data using SPSS version 22.0 (IBM Corp., Armonk, NY, United States). Categorical data were presented as frequencies and proportions, while continuous data following a normal distribution were described using means and standard deviations. Data that deviated from a normal distribution were represented using median and interquartile range. Differences in CPR KAP and self-efficacy among groups with varying demographic characteristics were compared using independent-samples *t*-test and one-way analysis of variance (ANOVA). We also explored the correlation among knowledge, attitude, practice, and self-efficacy using Pearson correlation analysis. Multiple linear regression analysis, using the stepwise entry method with a selection level of 0.05 and an elimination level of 0.10, was conducted to investigate the factors influencing public CPR KAP and self-efficacy. In this study, the dependent variable for multiple linear regression analysis was the total scores of public CPR KAP and self-efficacy, with statistically significant variables from single-factor analysis (independent-samples *t*-test and ANOVA) of participant sociodemographic data used as independent variables. All statistical tests were two-sided, and a *p*-value of ≤0.05 was considered statistically significant.

## 3 Results

### 3.1 Participant sociodemographic information

A total of 4,478 individuals participated in this questionnaire survey. Among them, 28 individuals declined to complete the questionnaire and exited. In the end, we received 4,450 survey responses from 31 provinces, autonomous regions, or municipalities across Mainland China. Of these, 3,340 (75.1%) were from Guizhou Province, Southwest of Mainland China. The participants ranged in age from 18 to 84 years old, with a median age of 28 years (interquartile range: 21, 40). Among the participants, 1,793 (40.3%) held a bachelor’s degree and above, and 1,603 (36.0%) were medical staff. Table 1 summarizes the sociodemographic characteristics of all 4,450 participants.

**Table 1:** Sociodemographic information of the participants and single-factor analysis of the factors influencing KAP and self-efficacy (*N* = 4,450).

Items	Number (%)	Statistical value	Knowledge (mean ± SD)	Attitude (mean ± SD)	Practice (mean ± SD)	Self-efficacy (mean ± SD)	Total score of KAP and self-efficacy (mean ± SD)
Gender							
Male	1,238 (27.8)		43.11 ± 13.25	45.07 ± 5.28	17.58 ± 2.47	13.93 ± 4.32	119.70 ± 21.34
Female	3,212 (72.2)		47.46 ± 12.90	46.39 ± 5.16	18.08 ± 2.39	14.54 ± 4.54	126.47 ± 21.42
		t^b^	−9.891	−7.514	−6.097	−4.131	−9.461
		p	<0.001	<0.001	<0.001	<0.001	<0.001
Age (year)^a^							
<28	2,126 (47.8)		45.76 ± 13.00	46.06 ± 5.41	18.01 ± 2.43	14.10 ± 4.57	124.71 ± 19.92
≥28	2,324 (52.2)		46.71 ± 13.24	45.99 ± 5.05	17.88 ± 2.41	14.62 ± 4.40	124.48 ± 23.05
		t^b^	−2.409	0.442	1.817	−3.910	0.351
		p	0.016	0.659	0.069	<0.001	0.726
Ethnicity							
Han	3,448 (77.5)		46.19 ± 13.42	46.05 ± 5.27	17.91 ± 2.46	14.40 ± 4.55	124.54 ± 22.02
Other	1,002 (22.5)		46.48 ± 12.10	45.94 ± 5.08	18.07 ± 2.26	14.27 ± 4.26	124.76 ± 20.15
		t^b^	−0.654	0.596	−2.028	0.795	−0.303
		p	0.513	0.551	0.043	0.427	0.762
Marital status							
Married	2,187 (49.1)		46.25 ± 13.87	46.01 ± 5.27	17.87 ± 2.49	14.60 ± 4.70	124.71 ± 22.90
Unmarried or divorced or widowed	2,263 (50.9)		46.26 ± 12.39	46.05 ± 5.19	18.02 ± 2.34	14.15 ± 4.26	124.47 ± 20.28
		t^b^	0.036	0.244	2.021	−3.322	0.382
		p	0.971	0.807	0.043	0.001	0.702
Education level							
Below bachelor’s degree	2,657 (59.7)		43.19 ± 12.46	44.92 ± 5.34	17.55 ± 2.47	13.46 ± 4.09	119.11 ± 20.22
Bachelor’s degree and above	1,793 (40.3)		50.79 ± 12.78	47.66 ± 4.59	18.53 ± 2.21	15.73 ± 4.71	132.71 ± 21.05
		t^b^	−19.751	−18.287	−13.869	−16.628	−21.47
		p	<0.001	<0.001	<0.001	<0.001	<0.001
Occupation							
Medical staff	1,603 (36.0)		55.64 ± 6.96	47.76 ± 4.17	18.75 ± 1.90	17.41 ± 2.96	139.56 ± 13.95
Other	2,847 (64.0)		40.97 ± 12.83	45.05 ± 5.50	17.49 ± 2.56	12.66 ± 4.29	116.16 ± 20.57
		t^b^	49.446	18.520	18.809	43.444	45.032
		p	<0.001	<0.001	<0.001	<0.001	<0.001
Place of residence							
City	1,701 (38.2)		48.87 ± 13.45	47.17 ± 4.92	18.34 ± 2.34	15.04 ± 4.97	129.41 ± 22.44
Countryside or town	2,748 (61.8)		44.64 ± 12.67	45.32 ± 5.28	17.70 ± 2.43	13.96 ± 4.11	121.60 ± 20.52
		t^b^	10.422	11.814	8.841	7.551	11.648
		p	<0.001	<0.001	<0.001	<0.001	<0.001
Personal health status							
Good	3,528 (79.3)		47.40 ± 12.77	46.33 ± 5.15	18.11 ± 2.34	14.60 ± 4.45	126.44 ± 21.15
General	876 (19.7)		42.02 ± 13.62	44.92 ± 5.24	17.34 ± 2.54	13.51 ± 4.54	117.78 ± 21.82
Poor/very poor	46 (1.0)		38.89 ± 12.72	43.89 ± 7.35	16.57 ± 3.09	12.78 ± 4.30	112.13 ± 22.53
		F^c^	68.282	29.778	44.481	24.113	66.005
		p	<0.001	<0.001	<0.001	<0.001	<0.001
Family health status							
No life-threatening illness	3,312 (74.4)		45.95 ± 13.10	45.87 ± 5.26	17.90 ± 2.41	14.30 ± 4.41	124.02 ± 21.50
Life threatening illness	1,138 (25.6)		47.13 ± 13.18	46.48 ± 5.11	18.06 ± 2.44	14.58 ± 4.71	126.25 ± 21.86
		t^b^	−2.612	−3.436	−1.874	−1.807	−3.004
		p	0.009	0.001	0.061	0.071	0.003
Living with an older adult over 60							
Yes	2,089 (46.9)		46.28 ± 13.11	46.09 ± 5.03	17.95 ± 2.39	14.51 ± 4.43	124.82 ± 21.48
No	2,361 (53.1)		46.24 ± 13.16	45.96 ± 5.39	17.94 ± 2.45	14.24 ± 4.54	124.38 ± 21.73
		t^b^	0.073	0.820	0.216	2.001	0.681
		p	0.942	0.412	0.829	0.045	0.496
Have heard of AED							
Yes	3,011 (67.7)		51.32 ± 10.68	47.14 ± 4.62	18.45 ± 2.14	15.74 ± 4.03	132.66 ± 18.33
No	1,439 (32.3)		35.64 ± 11.32	43.69 ± 5.63	16.87 ± 2.61	11.49 ± 3.99	107.70 ± 17.89
		t^b^	44.905	20.201	19.983	32.950	43.177
		p	<0.001	<0.001	<0.001	<0.001	<0.001
Have heard of CPR							
Yes	4,221 (94.9)		47.19 ± 12.66	46.27 ± 5.00	18.07 ± 2.31	14.55 ± 4.44	126.10 ± 20.84
No	229 (5.1)		28.84 ± 8.73	41.36 ± 6.73	15.53 ± 3.02	10.89 ± 3.87	96.64 ± 15.70
		t^b^	30.113	10.880	12.512	13.815	27.122
		p	<0.001	<0.001	<0.001	<0.001	<0.001
Ever encountered someone in need of CPR							
Yes	858 (19.3)		52.78 ± 9.57	46.97 ± 4.78	18.54 ± 2.19	16.54 ± 3.62	134.84 ± 17.51
No	3,592 (80.7)		44.69 ± 13.38	45.80 ± 5.30	17.79 ± 2.44	13.84 ± 4.51	122.14 ± 21.78
		t^b^	20.424	6.311	8.757	18.601	18.162
		p	<0.001	<0.001	<0.001	<0.001	<0.001
Ever performed CPR on others							
Yes	1,254 (28.2)		56.95 ± 6.01	48.29 ± 3.84	18.98 ± 1.84	17.99 ± 2.59	142.24 ± 12.49
No	3,196 (71.8)		42.05 ± 12.78	45.13 ± 5.42	17.53 ± 2.49	12.94 ± 4.26	117.66 ± 20.47
		t^b^	52.679	21.846	21.314	48.013	48.604
		p	<0.001	<0.001	<0.001	<0.001	<0.001
Ever trained in CPR							
Yes	2,710 (60.9)		54.19 ± 7.85	47.39 ± 4.42	18.52 ± 2.06	16.58 ± 3.31	136.70 ± 15.10
No	1,740 (39.1)		33.87 ± 9.66	43.89 ± 5.65	17.03 ± 2.63	10.92 ± 3.85	105.73 ± 15.98
		t^b^	73.471	21.875	19.902	50.446	65.226
		p	<0.001	<0.001	<0.001	<0.001	<0.001

a The median age of the subjects was 28 years.

b Independent-samples *t*-test.

c One-way analysis of variance.

SD, standard deviation; KAP, knowledge, attitudes, practices; CPR, cardiopulmonary resuscitation; AED, automated external defibrillator.

### 3.2 Knowledge of the public regarding CPR

As shown in Table 2, the level of understanding (clear and very clear) of the knowledge component regarding CPR ranged from 59.3 to 78.1%, with an overall average of 67.4%.

**Table 2:** Knowledge level of respondents regarding CPR (*N* = 4,450).

Items	Unclear and very unclear *n* (%)	Uncertain *n* (%)	Clear and very clear *n* (%)
Do you know why CPR should be performed as soon as possible when a person’s breathing and heartbeat suddenly stop?	323 (7.3)	651 (14.6)	3,476 (78.1)
Do you know how to assess whether the patient is breathing?	381 (8.6)	602 (13.5)	3,467 (77.9)
Do you know why it’s important to pat and call the patient loudly?	539 (12.1)	629 (14.1)	3,282 (73.8)
Do you know where to apply pressure during CPR?	659 (14.8)	598 (13.4)	3,193 (71.8)
Do you know how to recognize cardiac arrest in a patient?	660 (14.8)	626 (14.1)	3,164 (71.1)
Do you know the correct technique for applying pressure during CPR?	868 (19.5)	555 (12.5)	3,027 (68.0)
Do you know the recommended number of compressions per minute during CPR?	1,138 (25.6)	467 (10.5)	2,845 (63.9)
Do you know how to determine if patient rescue has been successful?	1,063 (23.9)	611 (13.7)	2,776 (62.4)
Do you know the appropriate depth of chest compressions during CPR?	1,217 (27.3)	486 (10.9)	2,747 (61.7)
Do you know the correct sequence for performing CPR?	1,298 (29.2)	459 (10.3)	2,693 (60.5)
Do you know the correct way to open the patient’s airway?	1,309 (29.4)	465 (10.4)	2,676 (60.1)
Do you know the recommended compression-to-ventilation ratio when performing on-site CPR as a single rescuer?	1,377 (30.9)	435 (9.8)	2,638 (59.3)
Average rate *n* (%)	899 (20.2)	506 (11.4)	3,000 (67.4)

CPR, cardiopulmonary resuscitation.

### 3.3 Attitudes of the public towards CPR

Positive attitudes (agree and strongly agree) ranged from 95.4 to 97.8%, with an average of 96.8%, as shown in Table 3.

**Table 3:** The public’s attitude towards CPR (*N* = 4,450).

Items	Disagree and strongly disagree *n* (%)	Uncertain *n* (%)	Agree and strongly agree *n* (%)
If I can provide timely first aid, I may save someone’s life.	25 (0.6)	72 (1.6)	4,353 (97.8)
I consider it a virtue to render aid to others.	29 (0.7)	74 (1.7)	4,347 (97.7)
I believe that learning CPR is necessary.	39 (0.9)	77 (1.7)	4,334(97.4)
I believe that first-aid equipment should be provided in public places such as schools and shopping malls.	21 (0.5)	100 (2.2)	4,329 (97.3)
Schools, communities and workplaces should regularly conduct CPR training.	27 (0.6)	99 (2.2)	4,324 (97.2)
CPR is not solely the responsibility of medical personnel. The public should learn CPR to assist their families or others when needed.	38 (0.9)	98 (2.2)	4,314 (96.9)
I think CPR should be taught in schools.	25 (0.6)	114 (2.6)	4,311 (96.9)
I am willing to participate in CPR knowledge and skills training.	50 (1.1)	107 (2.4)	4,293 (96.5)
I believe that chest compressions are an important life-saving measure for patients in cardiac arrest.	33 (0.7)	160 (3.6)	4,257 (95.7)
I am interested in learning CPR knowledge and skills.	42 (0.9)	162 (3.6)	4,246 (95.4)
Average rate *n* (%)	31 (0.7)	111 (2.5)	4,308 (96.8)

CPR, cardiopulmonary resuscitation.

### 3.4 Practices of the public with respect to CPR

The proportion of good practice (willing and strongly willing) ranged from 86.0 to 97.3%, with an average of 92.8%, as shown in Table 4.

**Table 4:** The public’s CPR practice (*N* = 4,450).

Items	Unwilling and strongly unwilling *n* (%)	Uncertain *n* (%)	Willing and strongly willing *n* (%)
When a family member, friend, or acquaintance suddenly stops breathing and their heart stops, are you willing to perform chest compressions for them?	24 (0.5)	96 (2.2)	4,330 (97.3)
When a family member, friend, or acquaintance suddenly stops breathing and their heart stops, are you willing to perform ventilation for them?	39 (0.9)	148 (3.3)	4,263 (95.8)
Would you be willing to perform chest compressions on a stranger who experiences sudden respiratory and cardiac arrest?	90 (2.0)	261 (5.9)	4,099 (92.1)
Would you be willing to perform ventilation on a stranger who experiences sudden respiratory and cardiac arrest?	185 (4.2)	438 (9.8)	3,827 (86.0)
Average rate *n* (%)	84 (1.9)	236 (5.3)	4,130 (92.8)

### 3.5 Self-efficacy of the public on CPR

The proportion of good self-efficacy (confident and extremely confident) ranged from 42.4 to 67.6%, with an average of 58.9%, as shown in Table 5.

**Table 5:** The public’s self-efficacy on CPR (*N* = 4,450).

Items	Not confident and not confident at all *n* (%)	Uncertain *n* (%)	Confident and extremely confident *n* (%)
If someone suddenly collapses, I am capable of correctly assessing their level of consciousness, heartbeat, and breathing.	699 (15.7)	743 (16.7)	3,008 (67.6)
I am capable of correctly administering ventilation to patients who are not breathing and have no heartbeat.	875 (19.7)	731 (16.4)	2,844 (63.9)
I am capable of correctly performing CPR on patients who are not breathing or have no heartbeat.	895 (20.1)	808 (18.2)	2,747 (61.7)
I am capable of properly using an automated external defibrillator (AED).	1,580 (35.5)	985 (22.1)	1,885 (42.4)
Average rate *n* (%)	1,010 (22.7)	819 (18.4)	2,621 (58.9)

CPR, cardiopulmonary resuscitation; AED, automated external defibrillator.

### 3.6 Single-factor analysis of factors influencing knowledge, attitude, practice and self-efficacy

The independent-samples *t*-test and one-way ANOVA indicated that gender, education level, occupation, place of residence, personal health status, have heard of AED, have heard of CPR, ever encountered someone in need of CPR, ever performed CPR on others, and ever trained in CPR had a significant impact on the KAP and self-efficacy scores (*p* < 0.05), as shown in Table 1.

### 3.7 Correlation analysis of knowledge, attitude, practice and self-efficacy

The results of Pearson correlation analysis indicated positive correlations between CPR knowledge and attitude (*r* = 0.513, *p* < 0.01), knowledge and practice (*r* = 0.492, *p* < 0.01), knowledge and self-efficacy (*r* = 0.781, *p* < 0.01), among others, as shown in Table 6.

**Table 6:** Correlation coefficients among CPR-related knowledge, attitude, practice, and self-efficacy (*N* = 4,450).

Items	Knowledge score	Attitude score	Practice score	Self-efficacy score
Knowledge score	1	–	–	–
Attitude score	0.513^**^	1	–	–
Practice score	0.492^**^	0.730^**^	1	–
Self-efficacy score	0.781^**^	0.415^**^	0.449^**^	1

** The correlation was significant at the 0.01 level (two-tailed). CPR, cardiopulmonary resuscitation.

### 3.8 Multiple linear regression analysis of the factors influencing KAP and self-efficacy

The variables found to have an effect on the total scores of KAP and self-efficacy in the single-factor analysis (independent-samples *t*-test and ANOVA) were included in the multivariate model. Using the total score of KAP and self-efficacy as the dependent variable, the 11 independent variables included were: gender (male = 1, female = 2), education (below bachelor’s degree = 1, bachelor’s degree and above = 2), occupation (other = 1, medical staff = 2), place of residence (countryside or town = 1, city = 2), personal health status (poor/particularly poor = 1, general = 2, good =3), family health status (no life-threatening disease = 1, life-threatening disease = 2), have heard of AED (no = 1, yes = 2), have heard of CPR (no = 1, yes = 2), ever encountered someone in need of CPR (no = 1, yes = 2), ever performed CPR on others (no = 1, yes = 2), ever trained in CPR (no = 1, yes = 2). The results showed that the regression model was statistically significant (*F* = 727.323, *p* < 0.001, adjusted R^2^ = 0.595), and the effect of the nine independent variables included in the model on the total scores of KAP and self-efficacy was statistically significant (*p* < 0.05), as shown in Table 7.

**Table 7:** Multiple linear regression analysis of the factors influencing the total score of KAP and self-efficacy (*N* = 4,450).

Variable	Partial regression coefficient (B)	Standard error (SE)	Standardized partial regression coefficient (beta)	*t*	*p*
Constant	74.767	0.798	–	93.664	<0.001
Ever trained in CPR	19.238	0.562	0.434	34.215	<0.001
Have heard of AED	8.968	0.528	0.194	16.982	<0.001
Ever performed CPR on others	6.863	0.608	0.143	11.284	<0.001
Have heard of CPR	7.142	0.994	0.073	7.184	<0.001
Occupation	4.307	0.573	0.096	7.522	<0.001
Personal health status	2.945	0.480	0.060	6.139	<0.001
Education	2.837	0.460	0.064	6.168	<0.001
Gender	2.401	0.471	0.050	5.098	<0.001
Ever encountered someone in need of CPR	1.310	0.568	0.024	2.308	0.021

KAP, knowledge, attitudes, practices; CPR, cardiopulmonary resuscitation; AED, automated external defibrillator.

## 4 Discussion

The aim of the present study was to investigate the KAP and self-efficacy regarding CPR among various population groups in mainland China. This survey was crucial in assessing the current state of CPR-related KAP and self-efficacy levels among the Chinese public, and to provide up-to-date evidence for enhancing the public’s first aid skills in the future.

The study findings indicated that the majority of the public had a good understanding of CPR knowledge, with an average mastery rate of 67.4%. However, previous studies conducted by Feng et al. (14) and Jin et al. (24) demonstrated that the public in Shanghai city and Hebei province had a lower grasp of CPR knowledge (36.1 and 33.5%, respectively) compared to the participants in our study. This discrepancy may be attributed to the inclusion of medical personnel (36.0%) in our study, who were not part of the previous studies. In recent years, the Chinese government and some organizations have emphasized the importance of training the public in first aid skills and have increased the intensity of such training (17, 18). While most of the public understands the significance of CPR, they may still have difficulty mastering certain technical aspects such as the depth of chest compressions, the correct sequence of CPR, the proper way to open a patient’s airway, the compression-to-ventilation ratio, and the number of compressions per minute. Therefore, it is necessary to further enhance the education and promotion of CPR knowledge and disseminate standardized CPR techniques to reduce mortality rates of OHCA patients and ensure precious rescue time for emergency personnel.

In this study, the public showed a positive attitude towards cardiopulmonary resuscitation (CPR), with over 96% of participants believed it was necessary to learn CPR, felt that learning CPR was not only the responsibility of medical staff but also the general public, and acknowledged that administering first aid in a timely manner could save lives. The majority of participants also felt that CPR should be included in school curricula and offered in public places, and expressed their willingness to participate in training programs to acquire relevant knowledge and skills. These findings are consistent with those of other studies (15, 19, 25), which suggest that the increasing number of OHCA and high mortality rates have led people to realize the importance of CPR for cardiac arrest patients, and to desire mastery of CPR knowledge and skills (26).

Most people showed good theorical knowledge about practice. More than 90% of the participants were willing to perform CPR on their relatives, friends, or strangers in the event of cardiopulmonary arrest. This may be due to the participants’ strong knowledge and positive attitude towards CPR, which led to good behavioral intentions. Additionally, influenced by traditional Chinese culture, benevolence and helping others are considered to be traditional virtues in China, resulting in most people being willing to offer free help to others. However, it is important to note that 14% of respondents indicated that they would not be willing to give ventilation to a stranger. The research findings by Jan Wnent and colleagues indicate that performing bystander CPR involving both chest compressions and ventilations is linked to a higher survival rate when compared to using chest compressions alone (27). Consequently, it holds paramount importance to incorporate both chest compressions and ventilations into CPR training courses and motivate trainees to utilize both techniques during real-life situations. Nevertheless, in cases where an individual has not received formal CPR training, compression-only CPR remains a viable and effective alternative in accordance with the latest CPR guidelines (4).

Compared to KAP, the public’s self-efficacy in properly performing CPR was significantly lower. While over 60% of the public can correctly assess consciousness, heartbeat, breathing, and perform CPR correctly, only 42.4% of the participants reported having confidence in the correct use of AED. This suggests that the public in China lacks confidence in performing CPR, particularly in correctly using AED. This may be due to the limited availability of AED in China and the inadequate allocation of AED resources in economically underdeveloped regions (28). The provision of high-quality CPR and early defibrillation is a critical link in the adult chain of survival, and early defibrillation concurrent with high-quality CPR is essential for survival in cases of sudden cardiac arrest caused by ventricular fibrillation or pulseless ventricular tachycardia (4). Therefore, expanding the accessibility of AED is a pressing issue that needs to be addressed.

This study also revealed a positive correlation among knowledge, attitude, practice, and self-efficacy, indicating a virtuous cycle among them. The better the public’s CPR knowledge, attitude, and practice, the stronger their self-efficacy and skill level, which is consistent with the findings of Liu et al. (20). Previous research has shown a strong association between CPR self-efficacy levels in the community and clinical outcomes, as well as the rate of bystander CPR (29). Therefore, it is recommended that in public CPR training, a comprehensive approach should be taken to improve the public’s KAP and self-efficacy, rather than focusing solely on one aspect.

The results of the multiple linear regression analysis showed that several factors have a significant influence on the public’s CPR-related KAP and self-efficacy, including ever having received CPR training, hearing about AED, performing CPR on others, hearing about CPR, occupation, personal health status, education level, gender, and encountering someone in need of CPR. Thus it can be seen the importance of CPR training is evident, as it is a crucial way to improve the public’s ability to perform CPR (30). Previous CPR training is associated with higher skills and survival-to-discharge rates, compared to no training (31). In addition, repeated CPR training has been shown to improve trainees’ attitudes and CPR quality, and increasing the number of training sessions can significantly increase willingness to initiate CPR and confidence in CPR skills, as well as improve chest compression depth, no-flow time, and mouth-to-mouth ventilation (MTMV) (30). It should be noted that, during CPR training for the general public, particular attention should be paid to the above-mentioned knowledge points with lower mastery rates. Tailored training materials should be used, incorporating easy-to-understand language and visual aids, according to the characteristics of the trainees. Utilizing diverse approaches such as a combination of theoretical learning and practical hands-on exercises, scenario simulations, and virtual simulations, helps individuals comprehensively grasp CPR-related knowledge and skills. Medical professionals scored higher on CPR KAP and self-efficacy, likely due to their professional educational background and on-the-job training. The better the individual’s health status, the higher their scores on CPR KAP and self-efficacy, as performing CPR can be physically demanding, and individuals in poor health may have difficulties performing this task and feel less willing to learn. The higher the education level, the higher the CPR KAP and self-efficacy score, which is a similar correlation found in other studies (13, 32). This may be because people with higher education are better at learning, can master CPR knowledge faster, and have more positive attitudes and better self-efficacy, ultimately leading to better practices. The scores of CPR KAP and self-efficacy in the female group were significantly higher than those in the male group, which is consistent with the findings of Feng et al. (14), potentially due to the relatively detailed knowledge memory of the female group. Encountering someone in need of CPR is also a significant factor affecting the public’s CPR KAP and self-efficacy, as witnessing a real cardiac arrest patient deepens the attention paid to CPR. It is recommended that the aforementioned factors be taken into consideration to further improve the popularity and effectiveness of CPR training in the future.

There are three main limitations to this study. Firstly, the sample was obtained using convenience sampling and snowball sampling. Although we collected data from 31 provinces, autonomous regions, or municipalities, 75.1% of the respondents were from Guizhou Province in Southwest China. Furthermore, the study was limited to adults aged 18 years and older. Consequently, the results may not fully represent the entire Chinese population, and the generalizability of our findings may be limited. Additionally, in this study, we only surveyed participants’ places of residence and did not inquire about their origins. Therefore, we analyzed only the influence of residency (city and countryside/town) on CPR KAP and self-efficacy. In future research, we will carefully consider this issue to conduct a more comprehensive analysis of the factors influencing public CPR KAP and self-efficacy. Secondly, this study was conducted as an online survey. The majority of participants were young individuals, with a median age of 28. Over one-third of the respondents were healthcare professionals, and more than 40% held a bachelor’s degree and above. These factors could indicate that the participants had higher motivation and opportunities to learn and practice CPR. Therefore, the data presented in this study may present a somewhat optimistic view of reality. Thirdly, the study relied on self-reported outcomes instead of on-site investigations and skill assessments. Consequently, the survey results may be susceptible to social desirability bias and could potentially be overestimated. To mitigate this bias, we stressed to participants that their responses would remain anonymous and confidential, with no right or wrong answers. Despite these limitations, the survey results from diverse participants representing different regions, professions, and educational backgrounds provide initial insights into the Chinese public’s knowledge, attitude, practice and self-efficacy related to CPR. This lays the foundation for future, more in-depth, and comprehensive research. It can contribute to guiding health education, training, and promotional efforts aimed at enhancing CPR awareness and skills among the Chinese public, ultimately increasing the chances of successful resuscitation for OHCA patients.

## 5 Conclusion

In summary, the Chinese public demonstrates good knowledge of CPR, positive attitude, and high willingness to perform CPR. However, there is still room for improvement in the mastery of some professional knowledge points related to CPR, such as the correct depth of chest compression, the proper sequence of CPR, the appropriate way to open the patient’s airway, the compression-to-ventilation ratio, and the number of compressions per minute during CPR. Additionally, the public lacks confidence in correctly performing CPR and using AED, which requires further improvement. It should be noted that knowledge, attitude, practice, and self-efficacy are interrelated and influence each other. Factors such as prior CPR training, hearing about AED, having performed CPR before, hearing about CPR, occupation, personal health status, education level, gender, and having encountered someone in need of CPR have a significant impact on the public’s KAP and self-efficacy. Therefore, focusing on these issues in future CPR training and education programs is important to improve the overall readiness and effectiveness of the public in responding to cardiac arrest.

## Data availability statement

The raw data supporting the conclusions of this article will be made available by the authors, without undue reservation.

## Ethics statement

The studies involving humans were approved by the Biomedical Research Ethics Committee of the Affiliated Hospital of Zunyi Medical University. The studies were conducted in accordance with the local legislation and institutional requirements. The participants provided their written informed consent to participate in this study.

## Author contributions

HG: Conceptualization, Formal analysis, Methodology, Writing – original draft. XL: Conceptualization, Formal analysis, Methodology, Writing – original draft. ZJ: Project administration, Writing – review & editing. SH: Conceptualization, Methodology, Writing – review & editing. XP: Formal analysis, Writing – original draft. JL: Conceptualization, Methodology, Writing – review & editing. QT: Conceptualization, Methodology, Writing – review & editing. LL: Conceptualization, Methodology, Writing – review & editing. MZ: Conceptualization, Methodology, Writing – review & editing. RH: Conceptualization, Funding acquisition, Methodology, Project administration, Writing – review & editing.
