# Supplementary material for: Knowledge, attitudes, practices, and self-efficacy of the Chinese public regarding cardiopulmonary resuscitation: an online cross-sectional survey
Source: Front Public Health. 2024 Feb 29;12:1341851. doi: 10.3389/fpubh.2024.1341851 (PMC10937378; doi:10.3389/fpubh.2024.1341851)
Supplement: Supplementary file 1 [file Table_1.DOCX]

Supplementary Material

**Supplemental material 1: questionnaire**

**Survey on the Current Status of Public Cardiopulmonary Resuscitation (CPR) Knowledge, Attitudes, Practices, and Self-Efficacy in China**

Dear Sir/Madam,

We are the Emergency Research Team at the Affiliated Hospital of Zunyi Medical University. We sincerely appreciate your valuable time in completing this survey. The purpose of this survey is to understand the knowledge, attitudes, parctices and self-efficacy related to Cardiopulmonary Resuscitation (CPR) among the Chinese public. This data will serve as a scientific basis for further improving the CPR awareness rate among the public. This survey may take approximately 5-10 minutes of your time. Your participation is anonymous, and all responses will be held in strict confidence. Your involvement in this survey will not affect you or your family in any way. Please respond truthfully based on your own perspectives. We greatly appreciate your support!

**Would you like to participate in this survey?**

□ Yes □ No

(If you choose "Yes," please continue; if you choose "No," you may exit the survey)

**Section One: Personal Information (Please mark "√" next to the selected answer or fill in the appropriate blank with your real response. For multiple-choice questions with explanations, please select as applicable.)**

1. Gender:

□ Male □ Female

2. Age (years): _______

3. Ethnicity:

□ Han □ Other Minority Ethnicity

4. Marital Status:

□ Unmarried □ Married □ Divorced □ Widowed

5. Education Level:

□ Middle School or Below □ High School/Technical School □ College/Associate Degree

□ Bachelor's Degree □ Master's Degree or Higher

6. Occupation:

□ Civil Servant □ Teacher □ Farmer □ Healthcare Professional □ Police Officer

□ Subway Employee, Bus Driver, Taxi Driver □ Tour Guide □ Laborer/Worker

□ Corporate Employee □ Retired □ Student □ Unemployed

□ Other (Please Specify): ________

7. Which provinces, autonomous regions, or municipalities are you from? _________

8. Your Place of Residence:

□ City □ Rural Area □ Town

9. Your Health Status:

□ Good □ General □ Poor □ Very Poor

10. Do any of your family members have life-threatening illnesses?

□ None

□ Cardiovascular diseases (e.g., congenital heart disease, coronary artery disease, heart attack, angina, sudden cardiac death, etc.)

□ Stroke (including ischemic stroke, hemorrhagic stroke, etc.)

□ Cancer (e.g., lung cancer, colon cancer, leukemia, breast cancer, etc.)

□ Uremia

□ Organ transplant recipient (e.g., heart transplant, kidney transplant, etc.)

□ Have a cardiac pacemaker implanted

□ Other: ___________________

11. Do you live with family members aged 60 or above?

□ Yes □ No

12. Have you heard of Automated External Defibrillators (AED)?

□ Yes □ No

(If you select "Yes," please continue to question 13; if you select "No," skip to question 14.)

13. Are you capable of using an AED?

□ Yes □ No

14. Have you heard of Cardiopulmonary Resuscitation (CPR)?

□ Yes □ No

(If you select "Yes," please continue to question 15; if you select "No," skip to question 16.)

15. What is the main source of your CPR knowledge? (Multiple choices allowed)

□ Social media like Tencent QQ, WeChat, etc.

□ Short video platforms like Tictok, Kuaishou, etc.

□ Television, movies, radio, etc.

□ Newspapers, books, promotional materials, etc.

□ Courses offered by schools

□ First aid knowledge lectures, special training

□ Family, friends, or professionals

□ Red Cross or hospital-sponsored public training

□ Other: ___________________

16. Have you ever encountered a situation where someone needed CPR outside of a hospital?

□ Yes □ No

17. Have you performed CPR on someone else?

□ Yes □ No

18. Have you attended CPR training?

□ Yes □ No

(If you select "Yes," please answer question 19; if you select "No," skip to question 20.)

19. Where did you receive CPR training? (Multiple choices allowed)

□ Medical schools □ Hospitals □ Emergency medical centers

□ Red Cross □ Community workers □ Other: ___________________

20. If you have not received CPR training, what is the main reason? (Multiple choices allowed)

□ Not relevant to me; don't want to participate

□ Want to learn but don't know where to learn

□ Too busy with work and studies; no time to learn

□ Don't think I'll ever need it, so don't want to learn

□ Physical condition doesn't allow

□ Other: ___________________

**Section Two: Knowledge (Please mark "√" in the appropriate box for each item based on your own understanding. Only one answer is allowed for each item.)**

| Items | Very clear | Clear | Uncertain | Unclear | Very unclear |
| --- | --- | --- | --- | --- | --- |
| 1. Do you know why CPR should be performed as soon as possible when a person's breathing and heartbeat suddenly stop? |  |  |  |  |  |
| 1. Do you know how to assess whether the patient is breathing? |  |  |  |  |  |
| 1. Do you know how to recognize cardiac arrest in a patient? |  |  |  |  |  |
| 1. Do you know why it's important to pat and call the patient loudly? |  |  |  |  |  |
| 1. Do you know where to apply pressure during CPR? |  |  |  |  |  |
| 1. Do you know the correct technique for applying pressure during CPR? |  |  |  |  |  |
| 1. Do you know the recommended number of compressions per minute during CPR? |  |  |  |  |  |
| 1. Do you know the appropriate depth of chest compressions during CPR? |  |  |  |  |  |
| 1. Do you know the correct way to open the patient's airway? |  |  |  |  |  |
| 1. Do you know the recommended compression-to-ventilation ratio when performing on-site CPR as a single rescuer? |  |  |  |  |  |
| 1. Do you know the correct sequence for performing CPR? |  |  |  |  |  |
| 1. Do you know how to determine if patient rescue has been successful? |  |  |  |  |  |

**Section Three: Attitudes (Please mark "√" in the appropriate box for each item based on your own understanding. Only one answer is allowed for each item.)**

| Items | Strongly agree | Agree | Uncertain | Disagree | Strongly disagree |
| --- | --- | --- | --- | --- | --- |
| 1. I believe that learning CPR is necessary. |  |  |  |  |  |
| 1. I believe that chest compressions are an important life-saving measure for patients in cardiac arrest. |  |  |  |  |  |
| 1. CPR is not solely the responsibility of medical personnel. The public should learn CPR to assist their families or others when needed. |  |  |  |  |  |
| 1. I am interested in learning CPR knowledge and skills. |  |  |  |  |  |
| 1. I am willing to participate in CPR knowledge and skills training. |  |  |  |  |  |
| 1. I think CPR should be taught in schools. |  |  |  |  |  |
| 1. I believe that first-aid equipment should be provided in public places such as schools and shopping malls. |  |  |  |  |  |
| 1. Schools, communities and workplaces should regularly conduct CPR training. |  |  |  |  |  |
| 1. I consider it a virtue to render aid to others. |  |  |  |  |  |
| 1. If I can provide timely first aid, I may save someone's life. |  |  |  |  |  |

**Section Four: Behavioral Intentions (Please mark "√" in the appropriate box for each item based on your own understanding. Only one answer is allowed for each item.)**

| Items | Strongly willing | Willing | Uncertain | Unwilling | Strongly unwilling |
| --- | --- | --- | --- | --- | --- |
| 1. When a family member, friend, or acquaintance suddenly stops breathing and their heart stops, are you willing to perform chest compressions for them? |  |  |  |  |  |
| 1. When a family member, friend, or acquaintance suddenly stops breathing and their heart stops, are you willing to perform ventilation for them? |  |  |  |  |  |
| 1. Would you be willing to perform chest compressions on a stranger who experiences sudden respiratory and cardiac arrest? |  |  |  |  |  |
| 1. Would you be willing to perform ventilation on a stranger who experiences sudden respiratory and cardiac arrest? |  |  |  |  |  |

**Section Five: Self-Efficacy (Please mark "√" in the appropriate box for each item based on your own understanding. Only one answer is allowed for each item.)**

| Items | Extremely confident | Confident | Uncertain | Not confident | Not confident at all |
| --- | --- | --- | --- | --- | --- |
| 1. If someone suddenly collapses, I can properly assess their consciousness, heartbeat and breathing |  |  |  |  |  |
| 1. In patients with no breathing and heartbeat, I can perform CPR correctly |  |  |  |  |  |
| 1. When encountering patients without breathing and heartbeat, I can perform artificial respiration correctly |  |  |  |  |  |
| 1. I can use an automated external defibrillator (AED) correctly |  |  |  |  |  |

**(The questionnaire is complete. Thank you once again for your support and assistance!）**

**中国公众心肺复苏知识、态度、行为及自我效能现状调查问卷**

**（Chinese）**

尊敬的先生/女士：

您好！我们是遵义医科大学附属医院急诊研究团队，非常感谢您在百忙之中填写这份调查问卷，本次调查的目的是了解我国公众对于心肺复苏（CPR）的知识、态度、行为以及自我效能等情况，为今后进一步提高公众的CPR普及率提供科学依据。本次调查可能会耽误您5-10分钟。本调查匿名进行，填答信息绝对保密，调查结果对您及家人无任何影响，请您根据自己的看法如实填写。感谢您的支持！

**您是否愿意参与本次调查：**□**愿意** □**不愿意**

**（愿意则继续填写，不愿意则退出）**

**一、基本情况**（请在选中的答案上打“√”，或把真实答案填写在相应空格上，除有说明可多选题外，其余均只选一项）

1. 性别： □男 □女
2. 年龄： 岁
3. 民族

□汉族 □少数民族

1. 婚姻

□未婚 □已婚 □离异 □丧偶

1. 教育水平：□初中及以下 □高中/中专 □大专 □本科 □硕士及以上
2. 职业

□公务员 □教师 □农民 □医务人员 □警务人员 □地铁职工、公交车司机、出租车司机 □导游 □工人 □企业人员 □退休人员 □学生 □无业/失业 □其他

1. 您来自哪个省/自治区/直辖市：
2. 您的居住地：□城市 □农村 □城镇
3. 您的健康状况 ：□良好 □一般 □差 □非常差
4. 您的家人是否患有威胁生命的疾病：

□无

□心血管疾病(例如先天性心脏病、冠心病、心肌梗死、心绞痛、猝死等)

□脑卒中(即中风、脑梗死、脑出血等)

□癌症(例如肺癌、大肠癌、白血病、乳腺癌等)

□尿毒症

□接受器官移植疾病(心脏移植、肾移植等)

□植入心脏起搏器

□其他

1. 您是否与60岁以上的家庭成员住在一起？ □是 □否
2. 您听说过自动体外除颤仪吗(AED)? □是 □否

**（选是，则继续填写第13题；选否，则跳到第14题）**

1. 您会使用自动体外除颤仪吗(AED)? □是 □否
2. 您听说过（知道）心肺复苏吗？ □是 □否

**（选是，则继续填写第15题；选否，则跳到第16题）**

1. 您心肺复苏相关知识的主要来源是？（可多选）

□腾讯QQ、微信等社交软件 □抖音、快手等网络平台 □电视、电影、广播等媒体

□报刊、书籍等宣传资料 □学校开展的课程 □急救知识讲座、专题培训

□家人、朋友或专业人士 □红十字会或医院等公益培训 □其他

1. 您曾遇到过需要在医院外进行心肺复苏的人吗？ □是 □否
2. 您是否给别人实施过心肺复苏？□是 □否
3. 您是否参加过心肺复苏培训？ □是 □否C

**（选择是，填写19题；选择否，则跳到第20题）**

1. 是什么机构、组织或个人提供的心肺复苏培训呢？

□医学院校 □医院 □急救中心 □红十字会 □社区工作者 □其他

1. 您没有接受过心肺复苏培训，您认为最主要的原因是：（可多选）

□跟自己无关，不想参加

□想学，但不知道在哪里学习

□工作和学习太忙，没时间学习

□学了可能用不到，所以不想学

□身体条件不允许

□其他

**二．知识**（请根据您自己的理解在每个条目后相应的空格里打“√”，每个条目只能选一个答案）

| **条目** | **非常清楚** | **清楚** | **一般** | **不清楚** | **非常不清楚** |
| --- | --- | --- | --- | --- | --- |
| 1. 您知道一个人呼吸心跳突然停止时，为什么要尽早进行心肺复苏吗? |  |  |  |  |  |
| 1. 您知道怎么判断患者是否有呼吸吗? |  |  |  |  |  |
| 1. 您知道怎么判断患者心跳停止吗? |  |  |  |  |  |
| 1. 您知道为什么要轻拍、大声呼叫患者吗? |  |  |  |  |  |
| 1. 您知道心肺复苏时手的按压位置吗? |  |  |  |  |  |
| 1. 您知道心肺复苏时正确的按压手法吗? |  |  |  |  |  |
| 1. 您知道心肺复苏时每分钟按压的次数吗? |  |  |  |  |  |
| 1. 您知道胸外按压的深度吗? |  |  |  |  |  |
| 1. 您知道正确开放患者气道的方法吗？ |  |  |  |  |  |
| 1. 您知道单人进行现场心肺复苏时，胸外按压和人工呼吸的比例吗？ |  |  |  |  |  |
| 1. 您知道心肺复苏正确的顺序吗? |  |  |  |  |  |
| 1. 您知道怎么判断患者抢救成功吗? |  |  |  |  |  |

**三．态度**（请根据您自己的理解在每个条目后相应的空格里打“√”，每个条目只能选一个答案）

| **条目** | **非常同意** | **同意** | **不清楚** | **不同意** | **非常不同意** |
| --- | --- | --- | --- | --- | --- |
| 1. 我觉得学习心肺复苏很有必要 |  |  |  |  |  |
| 1. 我认为胸部按压对心脏骤停患者来说是挽救生命的重要措施 |  |  |  |  |  |
| 1. 心肺复苏不只是医护人员的责任，公众应该学习心肺复苏术，以便在必要时帮助他们的家人或其他人 |  |  |  |  |  |
| 1. 我对学习心肺复苏知识与技能有很大的兴趣 |  |  |  |  |  |
| 1. 我愿意参加心肺复苏知识与技能培训 |  |  |  |  |  |
| 1. 我认为心肺复苏应列入学校的教学内容 |  |  |  |  |  |
| 1. 我认为在学校、商场等公共场所应配备急救设备 |  |  |  |  |  |
| 1. 学校、社区、单位应定期开展心肺复苏培训 |  |  |  |  |  |
| 1. 我认为对别人实施救护是一种美德 |  |  |  |  |  |
| 1. 如果我能够及时对他人进行急救，可能会挽救他的生命 |  |  |  |  |  |

**四．行为意向**（请根据您自己的理解在每个条目后相应的空格里打“√”，每个条目只能选一个答案）

| **条目** | **非常愿意** | **愿意** | **不清楚** | **不愿意** | **非常不愿意** |
| --- | --- | --- | --- | --- | --- |
| 1. 当您的家人、朋友等熟人突发呼吸心跳停止，您愿意为其做胸外心脏按压吗？ |  |  |  |  |  |
| 1. 当您的家人、朋友等熟人突发呼吸心跳停止，您愿意为其做人工呼吸吗？ |  |  |  |  |  |
| 1. 遇到突发呼吸心跳骤停的陌生人，您愿意为其做胸外心脏按压吗？ |  |  |  |  |  |
| 1. 遇到突发呼吸心跳骤停的陌生人，您愿意为其做人工呼吸吗？ |  |  |  |  |  |

| **条目** | **非常有把握** | **有把握** | **不清楚** | **没把握** | **完全没把握** |
| --- | --- | --- | --- | --- | --- |
| 1. 如果有人突然晕倒，我能正确评估其意识、心跳和呼吸 |  |  |  |  |  |
| 1. 遇到没有呼吸及心跳的患者，我能正确实施心肺复苏 |  |  |  |  |  |
| 1. 遇到没有呼吸及心跳的患者，我能正确实施人工呼吸 |  |  |  |  |  |
| 1. 我能正确使用自动体外除颤仪（AED） |  |  |  |  |  |

**五．自我效能**（请根据您自己的理解在每个条目后相应的空格里打“√”，每个条目只能选一个答案）

**（问卷到此结束，再次感谢您的支持和帮助！）**
